# Persistent organic pollutants and their effects on Arctic seabirds

**DOI:** 10.1186/1751-0147-54-S1-S8

**Published:** 2012-02-24

**Authors:** Geir Wing Gabrielsen

**Affiliations:** 1 Norwegian Polar Institute, Fram Centre, NO-9296 Tromsø, Norway

## 

Persistent organic pollutants (POPs) and their degradation and metabolic byproducts have been found in high levels in blood and tissues of several Arctic seabird- and mammal species (Glaucous gull (*Larus hyperboreus*), Ivory gull (*Pagophila eburnea*), Great skua (*Stercorarius skua*), Arctic fox (*Alopex lagopus*) and Polar bear (*Ursus maritimus*). The POPs include both old (PCBs, DDTs and CHLs) and new contaminants (polybrominated diphenyl ethers (PBDEs), perfluorinated compounds (PFCs) and polychlorinated naphthalenes (PCNs). While the contaminants which are subjected to global bans or restrictions are decreasing, new and emerging chemicals are increasing in Arctic birds and mammals. Several species are also exposed to contaminant metabolites (p, p-DDE, OH- and MeSO2-PCBs and OH-PBDEs) that, at times, are more bioactive than their precursors. Of high concern are the potential effects of the POPs and their metabolites in exposed seabird species. The present presentation summarizes recent studies on biological effects in relation to POP exposure in seabirds.

